# Repeated TLR7 activation induces cell type- and brain region-specific transcriptome changes in male mice

**DOI:** 10.1038/s41598-026-50920-5

**Published:** 2026-04-30

**Authors:** Marion M Friske, Riccardo Barchiesi, Nihal A Salem, Ruth L Allard, Anna C Dobre, Nicholas Rhyan, Wen Chen, R Dayne Mayfield

**Affiliations:** 1 Waggoner Center for Alcohol and Addiction Research, The University of Texas at Austin, 2500 Speedway, Austin, TX 78712 USA; 2 Department of Neuroscience, The University of Texas at Austin, 2500 Speedway, Austin, TX 78712 USA

**Keywords:** Molecular biology, Neuroscience

## Abstract

Alcohol consumption triggers neuroinflammation, potentially creating a feed-forward loop that increases drinking. Previous studies have shown that activation of the toll-like receptor 7 (TLR7) leads to escalated drinking. In this study, we aim to identify cell type-specific transcriptomic patterns underlying TLR7-induced neuroinflammation, potentially leading to escalated drinking. Therefore, male C57BL/6J mice were treated with the selective TLR7 agonist R848 every-other day for 20 days in total. After 10 treatment-free days, half of the cohort underwent two bottle-choice drinking; the other half was sacrificed and brains were collected for single-nucleus RNA-Sequencing (snRNA-Seq) in the medial prefrontal cortex (mPFC) and central amygdala (AMG). The AMG showed a greater number of differentially expressed genes (DEGs), primarily in inhibitory and excitatory neurons. Among glial cells, AMG astrocytes exhibited the greatest number of DEGs, which were involved in blood-brain barrier (BBB) regulation (e.g., *Cldn5*, *Mecom*, *Nrg1*), a finding supported by secondary validation using Xenium in situ spatial transcriptomics on AMG-containing sections. BBB-regulatory genes, including those in the Wnt signaling pathway (e.g., *Notch3*, *Top2a*, *Aldoc*), were altered across multiple cell types in both regions. Together with the alterations observed in neurons, these findings suggest that repeated TLR7 activation induces persistent BBB and neuronal dysregulation, potentially leading to TLR7-induced escalated drinking.

**Supplementary Information:**

The online version contains supplementary material available at 10.1038/s41598-026-50920-5.

## Introduction

Alcohol Use Disorder (AUD) is a major health and societal burden with limited and under-utilized treatment options^1,2^. Data from human postmortem brain tissue and rodent models indicate disruption of the innate immune system in alcohol dependence. Conversely, alterations in neuroimmune genes promote escalated drinking^3–6^. Among these, toll-like receptors (TLRs) are particularly implicated in the development and maintenance of the alcohol dependent state^7–9^. TLRs are pattern recognition receptors that are key drivers of the innate immune response. They act through the MyD88 signaling pathway (involving IRAK kinases and NF-κB) and the TRIF signaling pathway including interferon response factors (IRFs) and type I interferons (IFNs). Elevated TLR protein and mRNA levels have been observed in multiple brain regions of AUD donors compared to moderate drinking controls^10,11^. Several receptors, including TLR2, TLR3, TLR4, and TLR7, show positive correlations with lifetime alcohol consumption^12^. Manipulation of TLR2^13,14^, TLR3^8,15,16^ and TLR7^17,18^ regulate alcohol intake in rodents.

Persistent activation of TLR7 with the synthetic agonists R848 or R837 increases alcohol intake and preference in rodents using the two-bottle choice every other day (2BC-EOD) drinking model and operant alcohol self-administration^17,18^. Expression levels of downstream signaling genes of TLR7, such as *Irf7*, are also highly sensitive to alcohol^11,19–23^. TLR7 expression is significantly up-regulated in several regions of the human AUD brain and was correlated with lifetime alcohol consumption^10,11^. The molecular mechanisms linking TLR7-mediated neuroinflammation to escalated drinking, however, remain unclear. Given the sensitivity of downstream signaling genes, and the robust escalation of voluntary alcohol consumption following repeated TLR7 activation in mice, further investigation of the role of TLR7 in AUD and alcohol-related models is warranted.

Recent studies highlighted the medial prefrontal cortex (mPFC) and amygdala (AMG) as harboring neuroinflammatory signatures underlying alcohol-related behaviors, such as escalated drinking^24,25^. Their roles in excessive alcohol consumption and AUD are well-documented^26^. The human mPFC and similar structures in rodents exhibit distinct transcriptomic alterations due to alcohol exposure and AUD^27^. The AMG, a heterogeneous brain region, is implicated in alcohol-dependent behaviors, with the central AMG as a key regulatory region^28^. Prior research suggests brain region-specific signatures in TLR7-related transcripts^17^, but transcriptome-wide and cell type-specific expression patterns remain unclear.

Here, we profile cell-type specific transcriptomic changes in mPFC and AMG in mice following repeated TLR7 activation at the time point that has previously been reported to result in escalated drinking^17^. We identified a more than 4-times higher number of differentially expressed genes (DEGs) in the AMG with the majority of altered transcripts harbored in inhibitory neurons in both regions. We identified alterations in type I interferon signaling genes across multiple cells types indicating a persistent impact on down-stream processes directly mediated by TLR7. In addition, we identified cell type-specific and multi-cell type alterations of blood-brain barrier regulating genes with an emphasis on astrocytes and Wnt signaling.

## Results

### mPFC and AMG show distinct DEG patterns following repeated TLR7 activation

**Fig. 1:**
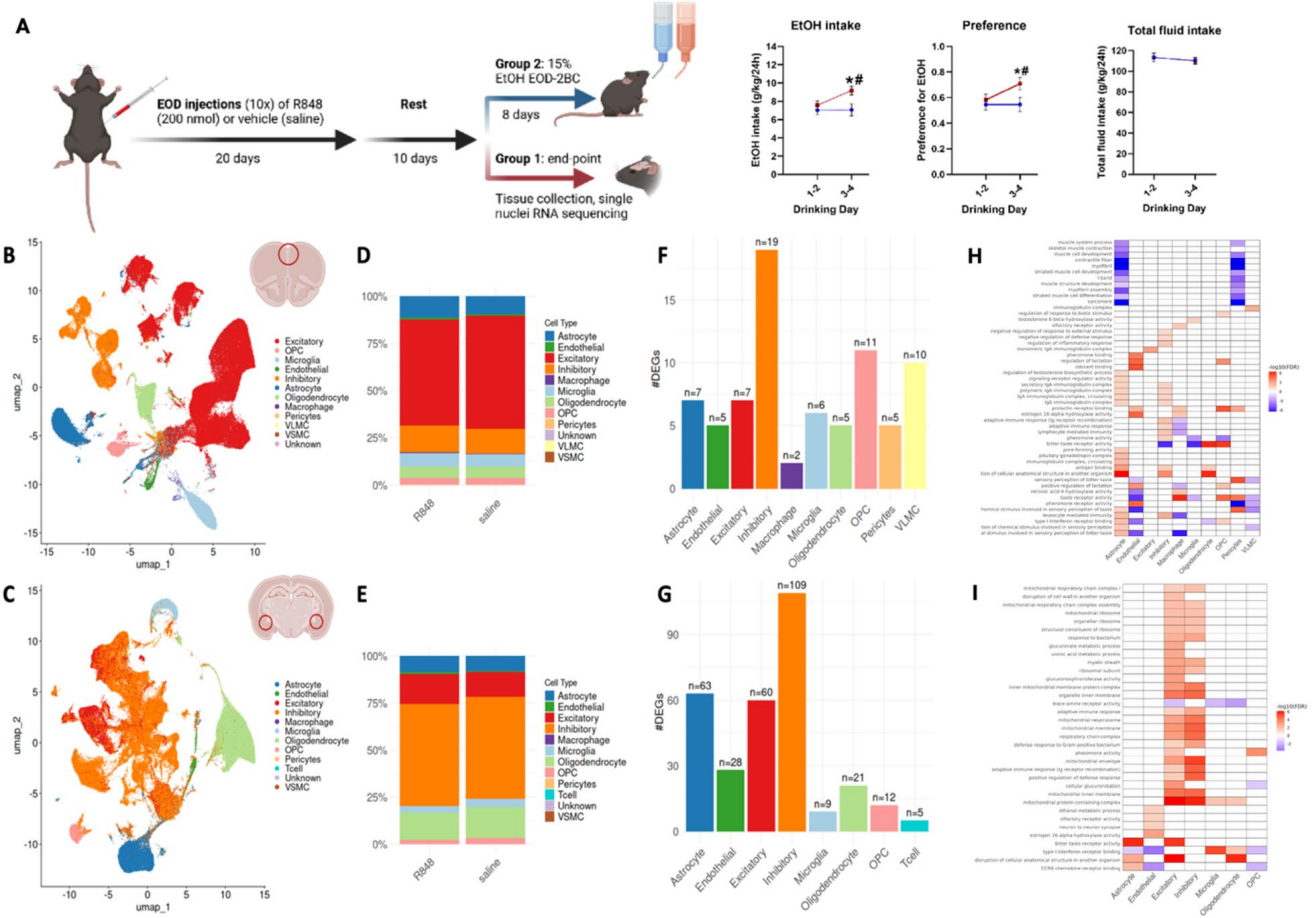
Cell type proportions and gene expression patterns in mPFC and AMG of R848-treated mice. (**A**) General experimental design and behavioral results. Male C57BL/6J mice were injected with R848 on an EOD schedule for 20 days. After 10 days of treatment-free phase, animals were sacrificed for snRNA-Seq. A second cohort underwent 2BC-EOD alcohol drinking for 8 days (4 drinking days) to confirm the escalated drinking phenotype as defined by a significant increase in alcohol drinking compared to controls. (**B**) and **C**) Uniform manifold approximation and projection (UMAP) plots displaying snRNA-Seq clusters from the mPFC and AMG, respectively. Cell-type proportions of all major cell types in the mPFC (**D**) and AMG (**E**) split by treatment. No significant treatment-specific effects on cell type composition were found. (**E**) Cell type proportion of all major cell types in AMG split by treatment. No significant treatment-specific effects on cell type composition were found. Bar graph showing the number of DEGs found in mPFC (**F**) (n_DEGs_=77; pval < 0.001) and AMG (**G**) (n_DEGs_=307; pval < 0.001). (**H**) Gene set enrichment analysis performed in major cell types of the mPFC including the top enriched activated and deactivated pathways. (**I**) Gene set enrichment analysis performed in major cell types of the AMG including the top enriched activated and deactivated pathways.

Male C57BL/6J mice were treated with R848 for 20 days. After a 10-day treatment-free phase, the animals were introduced to alcohol in a 2BC-EOD drinking paradigm (Fig. 1A, Group 2). Within the first four drinking days, the R848-treated mice escalated drinking (significant increase in alcohol consumption compared to controls) and showed significant preference for alcohol; total fluid intake was unaffected (Fig. 1A). While the C57BL/6J strain is known for escalated drinking and increased alcohol preference over time^29,30^, the exact time point these effects occur can vary. Therefore, our data suggests that chronic R848 treatment leads to a stronger effect on increase in voluntary drinking and preference.

We utilized snRNA-Seq in alcohol-naïve mice after the treatment-free period (Fig. 1A, Group 1) to profile cell type-specific transcriptomic patterns in the mPFC and AMG. Low quality nuclei with an expression of mitochondrial genes higher than 10% were filtered out of the count matrices, resulting in 132,837 mPFC and 129,365 AMG nuclei. Differential gene expression analysis was performed with a two-out-of-three approach using edgeR, limma, and DESeq2 methods with a threshold for differentially expressed genes (DEGs) of unadjusted p-value < 0.001. Since we observed transcriptomic profiles 10d after the last R848 treatment, we were expecting rather minor alterations in gene expression. Therefore, we aimed for an inclusive, but rigorous approach for defining DEGs when choosing the p-value cutoff.

We used supervised clustering to assign each cell to one of the 12 neuronal and 8 non-neuronal cell types as described in Bhattacherjee et al. (2019, 2023)^31,32^(Fig. 1B, Fig. S3A, Fig. S6A). Cell-type proportions were not significantly altered between groups, indicating that treatment did not affect overall cell type representation (Fig. 1D). Excitatory neurons comprised the majority of mPFC nuclei (58%) and astrocytes were the most abundant glial cell type (11%). Differential gene expression analysis identified 77 DEGs with 58.4% up-regulated (Fig. 1F, Table S1). Inhibitory neurons exhibited the highest number of DEGs (n_DEGs_=19), followed by oligodendrocyte precursor cells (OPCs; n_DEGs_=11) and vascular leptomeningeal cells (VLMCs; n_DEGs_=10).

In the AMG, each cell was assigned to one of the 20 inhibitory and 9 non-neuronal cell types as described in Hochgerner et al. (2023) and Yao et al. (2023)^33,34^(Fig. 1C, Fig. S3B, Fig. S6B). Consistent with mPFC results, cell type proportions were not significantly altered between groups (Fig. 1E). More than half of the nuclei (55.4%) were assigned to inhibitory neurons (Fig. 1E). Oligodendrocytes were the most abundant glial cell type (18%). These cell type proportions were consistent with prior studies in the rodent AMG^33,35^. Differential gene expression analysis revealed 307 DEGs (Fig. 1G, Table S2) with 86% up-regulated. The most DEGs were found in inhibitory neurons (n_DEGs_=109), astrocytes (n_DEGs_=63), and excitatory neurons (n_DEGs_=60).

Rank-rank hypergeometric overlap (RRHO) was performed to identify concordantly expressed genes per cell type across both brain regions with ranked gene lists considering fold-change and p-value as inputs for the analysis (Fig. S4). Up-regulated transcripts were more concordant than down-regulated genes. The strongest effect of concordant gene expression was found in inhibitory and excitatory neurons. Microglia and astrocytes were the only cell types with high concordance in both directions.

Cell-cell communication focusing on ligand-receptor pairs across major cell types in both brain regions revealed a clearer signal in the AMG with two factors showing significant changes between the treatment groups (Fig. S5). Factor 2 showed lower context loading scores in the R848 group (*p* = 0.042), indicating reduced contribution of this program in the R848-treated mice. (Fig. S5A,** B**). Pathway enrichment analysis of contributing ligand-receptor pairs resulted in synaptic signaling and synapse properties. Strongest loadings from sender cell types included endothelial cells, oligodendrocytes, and microglia and receiver loadings concentrated in excitatory and inhibitory neurons (Fig. S5C,** D**). Factor 3 showed higher context loading scores in the R848 group (*p* = 0.031). Sender loadings are dominated by astrocytes and, secondarily, OPCs across a broad set of received cell types (Fig. S5A,** B**). Pathway enrichment analysis pointed towards neurogenesis and neuronal development as characterizing pathways of these ligand-receptor pairs (Fig. S5C, D). Notably, among all pathways enriched in the 5 identified factors, the only down-regulated pathway is “regulation of canonical Wnt signaling pathway” in factor 1, which is mostly represented by oligodendrocytes interacting with microglia and endothelial cells and is up-regulated, though not significant, in the R848 group (Fig. S5).

### Microglia show altered subtype composition and interferon signaling

**Fig. 2:**
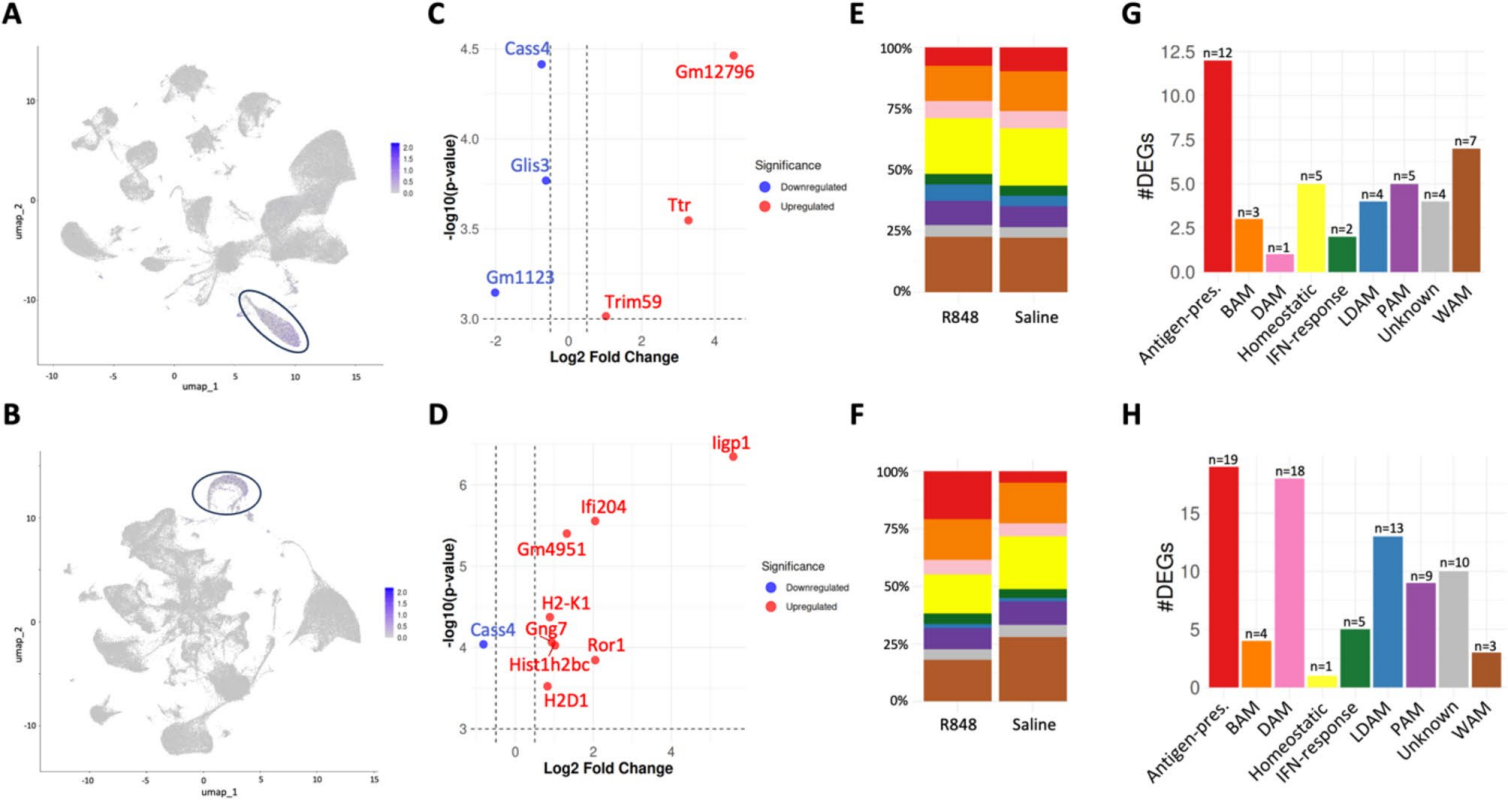
Expression profile of mPFC and AMG microglia. *Tlr7* expression profile in mPFC (**A**) and AMG (**B**). Volcano plots of mPFC (**C**) and AMG (**D**) microglia depicting all DEGs at *p* < 0.001). **E**) Proportion of microglia subtypes in the mPFC (**E**) and AMG (**F**). DEGs per microglia subtype in the mPFC (**G**) and the AMG (**H**). BAM = border-associated microglia, DAM = disease-associated microglia, LDAM = lipid droplet-accumulating microglia, PAM = proliferative-region-associated microglia, WAM = white matter-associated microglia.

Our data confirmed predominant expression of *Tlr7* in microglia^35–38^ with 52% of all *Tlr7*-expressing cells in mPFC and 58% in AMG (Fig. 2A & B). The mPFC harbored 6 DEGs (7.8% of all mPFC DEGs; Fig. 2C, Table S1), including two IFN-stimulating genes; *Glis3* and *Trim59*. In the AMG, 9 DEGs were detected (2.9% of all AMG DEGs; Fig. 2D, Table S2). We imported the DEGs derived from microglia into a recently published dictionary of immune responses to cytokines^39^ to perform cytokine response analysis. This immune response enrichment analysis considers gene set scores based on the sum of the normalized expression values and compares those scores using the Wilcoxon rank-sum test. This analysis on AMG microglia DEGs revealed significant enrichment in type I IFNs (e.g., IFNα1, IFNβ, IFNγ) and cytokine responses (e.g., *IL15*, *IL18*), and evidence, though not significant, for similar mechanisms in mPFC (Fig. S7).

mPFC microglia subtype proportions did not change significantly between treatments (Fig. 2E). In the AMG, R848-treated mice exhibited an increase in antigen-presenting microglia (FDR < 0.1) and a decrease in white matter-associated microglia (FDR < 0.1) (Fig. 2F), which may be linked to inflammation-induced alterations in myelination. IFN-response microglia were slightly increased in the AMG, but not in mPFC. In both regions, antigen-presenting HLA microglia had the most DEGs (Fig. 2G and H, Table S3 and S4).

In addition, the oligodendrocyte cluster with the highest number of DEGs revealed a high number of microglia marker genes (e.g., *Inpp5d*, *Siglech*, *Cx3cr1*) (Fig. S8). DEGs point towards a reactive oligodendrocyte population including up-regulated cytokine signaling (e.g., *Abcb7*, *Camk4*, *Phactr1*) and neuron-glia communication (e.g., *Cntn3*, *Cntnap5a*, *Tenm1*). These alterations might represent a compensatory mechanism of oligodendrocytes to the reduction in white matter-associated microglia.

RRHO focusing on concordant gene expression in mPFC and AMG showed enrichment in phagocytosis regulation, and leukocyte activation, and differentiation in down-regulated genes. Commonly up-regulated genes are enriched in purine biosynthesis, oxidation/oxidative phosphorylation, and ATP synthesis (Fig. S4A).

### Inhibitory neurons show the highest number of DEGs after repeated TLR7 activation

Inhibitory neurons exhibited the most DEGs in both regions (mPFC: 19 DEGs; 24.6%, AMG: 109 DEGs; 35.5%) (Fig. 1F, G). In the mPFC, these genes were involved in the innate immune system, such as IFN-signaling genes (e.g., *Ifi27*, *H3f3a*, *Prkd1*), anti-viral support genes (*Dock5*, *Ifi27*), and type I IFN regulation (*Blk*). In the AMG, DEGs were associated with immune regulatory processes, including stimulation of T-cell proliferation (*Acsbg1*, *B2m*), host defense (*Bcas1*, *Bst2*, *C4b*, *Ctsh*), IFN-γ-related mechanisms (*C4b*, *Cldn5*, *Il33*, *Ctss*), and TLR-interaction (*Slco1a4*, *Ly86*, *AU020206*, *AW112010*, *Cryab*). Dysregulation of neuronal functions i.e., neuronal signaling (*B2m*, *Glul*) and myelination (*Bcas1*, *Cldn11*) was consistent with TLR7’s role in neuronal development and morphology^40^.

Differential gene expression analysis revealed 54 DEGs across six mPFC subtypes and 752 DEGs in 20 AMG subtypes (Fig. S6, Table S5 and S6). DEGs across these subtypes were predominantly involved in neuronal development, synapse integrity, and cell adhesion (e.g., *Cntn4*, *Cntn6*, *Sorcs3*, *Npas3*, *Pdzrn4*). Furthermore, DEGs were linked to the TLR7-stimulated transcription factor IRF7, including *Nid2* (up-regulated in mPFC Vasoactive intestinal polypeptide-expressing interneurons (Vip)) and *Pfn1* (up-regulated in AMG Vip). Both regions showed dysregulated IFN-α signaling pathway transcripts e.g., *Ifi204* (up-regulated in AMG Somatostatin-expressing interneurons (Sst)), *Pdlim2* (up-regulated in AMG Sst), and *S1pr1* (up-regulated in AMG Vip). Pathway enrichment analysis in mPFC inhibitory neurons revealed enrichment in inflammatory pathways such as adaptive immune response, leukocyte mediated immunity, and IgA complex activity (Fig. 1H). In the AMG, activated pathways pointed towards cellular respiration and mitochondria function (Fig. 1I).

In the mPFC, the subtypes exhibiting the highest number of altered transcripts are Lamp5/Pax6 (n_DEGs_= 14) and Vip neurons with DEGs (n_DEGs_= 14). Comparison to our previous snRNA-seq study on CIE-treated mice^38^ confirmed a high number of DEGs in inhibitory neurons and revealed seven overlapping DEGs and six differentially expressed in inhibitory neurons in both datasets, namely *9630014M24Rik*, *Cntnap5c*, *Htr4*, *Smad7*, *Sorcs3*, *Zfyve9.* The cell type harboring the highest number of DEGs in the CIE study was inhibitory C corresponding to Lamp5/Pax6/Vip interneurons. In the AMG, Lamp5-Pdlim5 neurons revealed the highest number of DEGs (n_DEGs_= 163), which were predominantly enriched in cell adhesion and neuronal signaling (e.g., *Cadm2*, *Csmd1*, *Epha6*, *Pcdh15*). Even though our previous snRNA-seq study in human postmortem brain^41^ revealed the majority of transcriptomic alterations in glial cells, neuronal signatures overlapped with our study in enriched functional categories, such as synapse organization, and on a gene level with common DEGs such as *Negr1*, *Npas3*, *Oxr1*, *Thrb*. Notably, all those common genes are up-regulated in human postmortem and down-regulated in R848-treated mice. Even though there are overlapping transcriptomic signatures in the mPFC of the human postmortem brain and this dataset, the general transcriptomic profiles point more towards altered neuronal function in the human brain, whereas in the R848, the major signature is enriched in neuroimmune response.

CIE treatment in rats revealed a majority of DEGs in inhibitory neurons with most alterations found in Prkcd neurons. We observed 26 genes commonly dysregulated in our R848 AMG dataset and the CIE rats with 21 concordantly up-regulated DEGs and none commonly down-regulated. Pathway enrichment pointed towards neuroinflammatory pathways such as Interleukin-2 signaling, NGF signaling, and G alpha I pathway. A recent human postmortem AUD single nucleus multi-omics study in the AMG^42^, reported the highest number of DEGs and cis-regulated gene expression in inhibitory neurons. Cis-regulatory signatures also significantly correlated with AUD traits and other psychiatric disorders. Comparison with our CeA data revealed 208 unique common DEGs in inhibitory neurons with 75% concordantly up- and 6% down-regulated DEGs.

RRHO of concordantly down-regulated genes in both brain regions show enrichment in regulation of membrane potential, cilium organization and assembly, and microtubule- and cytoskeleton-related processes. Commonly up-regulated genes are enriched in ATP synthesis, respiratory processes, and purine biosynthesis (Fig. S4B).

### Astrocytes exhibit the greatest number of DEGs among glial cells

**Fig. 3:**
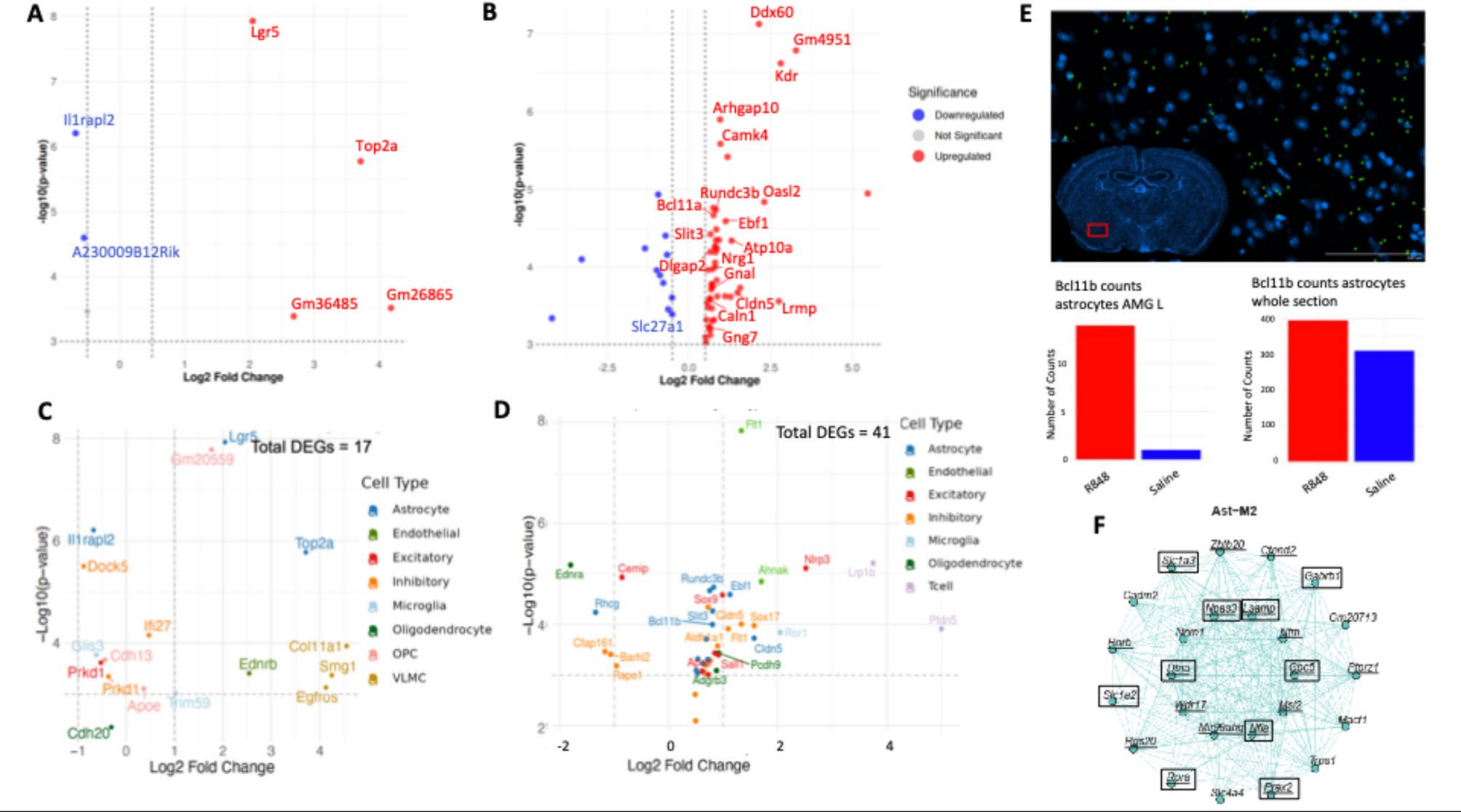
Gene expression profiles in astrocytes reveal persistent neuroinflammatory signatures and alcohol-sensitive genes. Volcano plot of mPFC (**A**) and AMG (**B**) astrocytes. Dashed lines highlight the fold-change (EdgeR) of +/-0.5. (**C**) Wnt signaling transcripts identified in the mPFC. DEGs are highlighted by cell type indicating an overall, non-cell type-specific mechanism. Dashed lines highlight a fold-change (EdgeR) of +/-1.0. (**D**) Wnt signaling transcripts identified in the AMG. DEGs are highlighted by cell type indicating an overall, non-cell type-specific mechanism. Genes are labeled if fc > 0.8. (**E**) Secondary validation using Xenium spatial transcriptomics focusing on the expression of *Bcl11b* in AMG-containing sections and AMG-specific subset. Green dots in the zoomed-in image of the Xenium AMG slice indicate Aldh1l1 expression as a marker for astrocytes. (**F**) The most significant module from hdWGCNA in the astrocytes (adj.p.val = 5.5 × 10-51; average log2 fold change= -0.26; Fig. S11C) showed substantial overlap with modules previously identified in both alcohol dependent mice (Salem et al. (2025), underlined) and humans (Warden et al. (2025), black boxes).

In mPFC-derived astrocytic nuclei, the 7 identified DEGs (Fig. 3A, Table S1) were not contributing to one specific pathway. In the AMG, astrocytes were the second most affected cell type. DEGs were associated with IFN-α signaling (e.g., *Atp10a*, *Ddx60*, *Oasl2*), calcium signaling (e.g., *Caln1*, *Camk4*, *Gng7*, *Lrmp*), neuronal development (e.g., *Arhgap10*, *Bcl11a*, *Ebf1*, *Kdr*), synaptic plasticity and neuronal signaling (e.g., *Dlgap2*, *Gm4951*, *Gnal*, *Nrg1*), and BBB-regulation (e.g., *Slc27a1*, *Nrg1*, *Cldn5*) (Fig. 3B, Table S2). Gene set enrichment analysis revealed activation of immunoglobin-related pathways and general immune responses, including type I interferon receptor binding in PFC (Fig. 1H), and CCR6 receptor binding and type I interferon receptor binding in AMG (Fig. 1I).

In astrocyte subclusters, BBB-related markers and DEGs were identified in both regions (Fig. S7, Table S7 and S8). Among those, genes contributed to both, functional (e.g., *Kdr*, *Slc27a1*, *Slit3*) and structural (e.g., *Cldn5*, *Fras1*, *Hmcn1*) BBB regulation. The global, non-specific enrichment of Wnt signaling genes (Fig. 3C and D), a BBB-regulatory mechanism, further supports the hypothesis that TLR7-induced BBB leakage contributes to escalated drinking. However, at this stage, the BBB dysregulation might predominantly emerge from astrocytes resulting in similar but less signatures in other cell types. In addition, concordantly down-regulated genes in mPFC and AMG are strongly enriched in Wnt signaling, whereas commonly up-regulated genes are enriched in cellular respiration, oxidative phosphorylation, and ATP synthesis (Fig. S4C).

### Spatial transcriptomics validates gene expression profiles in AMG astrocytes

The Xenium spatial in situ platform was utilized to profile AMG-containing coronal sections from the same R848- and saline-treated cohort used for snRNA-seq. This approach enabled cell type-specific validation with additional spatial context. The AMG exhibited a substantially higher number of DEGs with a particularly high number of TLR7-activated neuroinflammation and BBB-genes in astrocytes (Figs. 1 and 3). Consequently, we focused on astrocyte DEGs using two approaches: (1) an AMG-focused subset of the entire brain section, exclusively containing cells from the central AMG (termed “AMG crop”), to compare snRNA-Seq data to Xenium expression profiles, and (2) the entire AMG-containing section to identify consistent alterations across multiple brain regions. Of the 63 DEGs in AMG astrocytes (Fig. 3B), 24 were included in the Xenium 5 K mouse pan-tissue panel. Astrocyte-specific alterations, such as the inflammatory genes *Bcl11b*, *Nrg1* and *Hist1h2bc* were confirmed in astrocytes across the entire section (Fig. 3E, Fig. S10). Astrocytes co-expressing *Nrg1* and *Hist1h2bc* were increased in R848 treated mice (Fig. S10). Differential gene expression analysis validated numerous DEGs with BBB-related functions (e.g., *Nrg1*, *Kcnma1*, *Slc1a3*, *Sorbs1*).

### hdWGCNA analysis in AMG astrocytes identified common signatures previously linked to AUD

High dimensional weighted gene co-expression network analysis (hdWGCNA) was performed to identify common astrocytic gene networks with co-expression patterns overlapping with alcohol-related phenotypes as potential mechanism of escalated drinking. The hub genes identified in AMG astrocytes highlighted co-expressed transcripts involved in neuroinflammation, calcium signaling, and BBB-regulation, including Wnt signaling.

We identified genes in modules Ast-M4 and Ast-M5 that were previously reported as DEGs in a transcriptome-wide analysis of enriched astrocytes from mice with chronic alcohol drinking^19^(Fig. S11A). In Ast-M2, 17/25 hub genes overlapped with an astrocyte module dysregulated in CIE-treated mice^38^ and 10/25 hub genes overlapped with an astrocyte module dysregulated in individuals with AUD, compared to moderate drinkers^41^(Fig. 3F, Fig. S11).

## Discussion

This study investigated the molecular mechanisms underlying repeated TLR7-activation by the synthetic agonist R848. Our findings highlight persistent cell type- and brain region-specific adaptations following TLR7-induced inflammation including: (**i**) repeated R848 administration leads to increased alcohol intake and preference. (**ii**) snRNA-Seq revealed a more than 4-fold higher number of DEGs in the AMG, while in both regions the most DEGs were found in inhibitory neurons. (**iii**) Transcriptomic patterns suggest dysregulation of BBB-related mechanisms in both, a cell type-specific and non-specific manner. These mechanisms may promote excessive alcohol intake and preference.

### In both brain regions, most DEGs were found in inhibitory neurons

DEGs were globally involved in IFN and TLR7 signaling pathways. This was expected as TLR7 activation results in IFN production via IRF7^43^. Several of these genes also have been linked to alcohol, such as *Oasl2*, *Top2a*, and *Cryab*. The greatest number of DEGs was found in inhibitory neurons in both, mPFC and AMG. Subclustering revealed enrichment of DEGs in mPFC Lamp5/Pax6 and Vip neurons. Lamp5/Pax6 neurons exhibit neuroglia-like morphology characterized by stellate dendrites, numerous primary dendrites, and extensive axon branching^44^. Their involvement in neuronal network formation and maintenance suggests a role in memory formation. Vip neurons are involved in reward anticipation and dysregulation may impact the rewarding effects of alcohol^45^. Comparison to previous snRNA-seq studies in mouse models of AUD and human postmortem brain revealed a general high sensitivity of inhibitory neurons with more consistent signatures across the AMG datasets, suggesting that the R848-induced alterations in the AMG might be more translatable to AUD phenotypes. However, further functional investigation is needed to understand the common transcriptomic profiles of TLR7-mediated neuroinflammation and AUD.

Comparison to our previous snRNA-Seq study of CIE-treated mice^38^ confirmed a high number of DEGs in inhibitory neurons and revealed overlapping DEGs. Out of the 7 common DEGs identified in these mPFC datasets, 6 were in inhibitory neurons in both datasets, namely *9630014M24Rik*, *Cntnap5c*, *Htr4*, *Smad7*, *Sorcs3*, *Zfyve9*. The subtype with the highest number of DEGs in CIE mice was the same in this study.

Transcriptomic signatures in inhibitory neurons and the subgroup-specific analyses indicate dysregulation of major neuronal functions, including neuronal development, synapse integrity and cell adhesion, and a persistent activation of type I interferon signaling. These genes link neuroimmune activation via TLR7 to neuronal dysfunction, potentially causing altered behaviors such as escalated drinking.

DEGs identified in neurons revealed alterations in well-known markers for oligodendrocytes, such as *Apoe*, *Mbp*, and *Olig1*. It was observed across several cell types that cell type-specific markers appeared differentially expressed in atypical cell types. However, for most of those genes, either their fold-change was low (FC < 1.0) or the average expression was close to sequencing detection limits.

### AMG microglia show changes in subtype composition

Few DEGs were identified in microglia from either brain region. A possible explanation might be that while the majority of TLR7 is expressed in microglia, the predominant role of microglia might lie in the acute response to TLR7 activation but not in its persistent effects. We have previously shown that microglia are critical to dependence-induced escalation of alcohol intake^25^, but do not regulate voluntary alcohol consumption in non-dependent mice^46^. The role of microglia in TLR7-induced escalation of alcohol intake remains to be determined. We hypothesize that microglia may play a key role in inducing TLR7-mediated neuroinflammation, eventually leading to escalated drinking. However, at the time point we observe escalated drinking in this treatment, microglia may be less important than other cell types harboring larger transcriptomic alterations. Our previous study focused on the targeted expression of TLR7-related transcripts across different time points of chronic and acute R848 treatment, suggested that *Tlr7* may be predominantly altered shortly (8 h) after R848 administration, while down-stream components of this pathway, mainly *Irf7*, remain dysregulated persistently^17^.

Microglia subclustering revealed a brain region-specific shift in subtype representation, with a significant increase in antigen-presenting microglia and decrease in white matter-associated microglia in the AMG. Specifically, the decrease in white matter-associated microglia together with the inflammatory signatures in R848-treated oligodendrocytes may induce long-lasting alterations in neuronal myelination which could impact behavior. white matter-associated microglia are mostly involved in clearing myelin debris and their dysfunction is associated with aging and neurodegeneration^47^. They respond to neuroinflammatory processes and increased type I IFN expression has been associated with hyperactivated microglia in white matter^48^.

### Astrocyte DEGs were involved in neuronal regulation and alcohol-sensitive signatures

Several astrocyte DEGs represented important regulators of neuroimmune or calcium signaling pathways, suggesting a link between neuroimmune activation and dysregulated prevalent astrocyte functions. Previous studies show that calcium signaling in astrocytes regulates drinking and the sedative effects of alcohol in mice^49^. Comparison with our previous study on astrocyte-specific gene expression after long-term voluntary alcohol consumption in mice^19^ revealed overlapping signatures with the astrocyte expression patterns after repeated TLR7 activation. Even though *Tlr7* is predominantly expressed in microglia, astrocytes interact with TLR7 during immune activation, presumably via TLR7-mediated monocyte infiltration^50–52^. Measurable levels of *Tlr7* are expressed in astrocytes and activation of TLR7 in microglia can lead to an induction of inflammatory responses in astrocytes including the activation of reactive astrocytes^53^.

In two separate studies^38,41^, we identified an astrocyte-specific module of co-expressed genes in alcohol dependent mice and humans. This WGCNA module shares numerous hub genes with the Ast-M2 module in AMG astrocytes such as the transcription factor *Npas3*, the transmembrane proteoglycan *Gpc5*, and *Slc1a3*, which are involved in alcohol-related behaviors^54,55^(Fig. S10). While the modules were upregulated in alcohol dependent mice and humans, and downregulated here, interpreting directionality between different paradigms and time points is complicated.

**Fig. 4:**
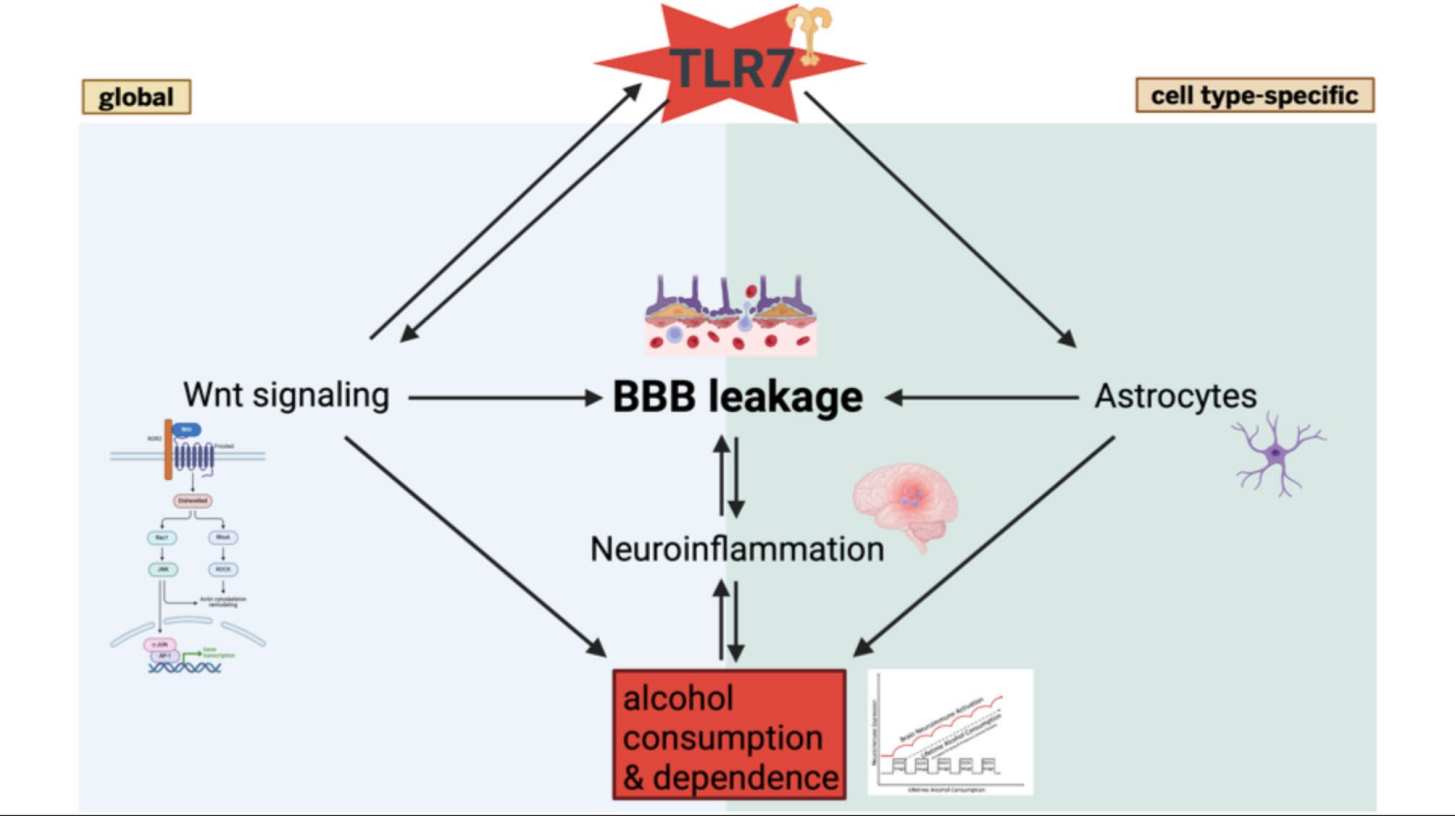
Proposed mechanism of TLR7 activation leading to BBB leakage and increased alcohol consumption. Global effect across multiple cell types suggests alterations in Wnt signaling pathways. Furthermore, cell type-specific mechanisms in astrocytes may exacerbate the effects on BBB leakage and alcohol-related outcomes. These pathways and their potential interaction may lead to BBB leakage and neuroinflammation as well as escalated alcohol consumption and alcohol dependent behaviors.

Our data indicate that repeated R848 administration links TLR7-mediated neuroinflammation to impaired astrocytic functions, including neuronal maintenance. These mechanisms may contribute to excessive alcohol consumption (Fig. 4).

### Chronic TLR7 activation causes persistent BBB dysregulation in a cell type- and non-specific manner

Manual annotation of DEGs to functional pathways across all granular cell types highlighted an involvement in neuroimmune signaling genes, primarily involving type I IFNs and IFN-γ, and DEGs associated with BBB-regulatory mechanisms, including Wnt signaling. GSEA revealed predominantly an enrichment in type I IFN signaling and other inflammatory pathways. On a cell type-specific level, astrocyte DEGs were particularly involved in BBB regulation. Spatial Transcriptomics validating the gene expression signatures in astrocytes across AMG-containing sections (Fig. 3E) suggested a potential non-region-specific effect of these signatures. TLRs have BBB-regulatory properties^56,57^. TLR7 activation during fetal development leads to BBB leakage^58^. Inflammation in the periphery via e.g., viral infection or pain, induces BBB disruption by over-expression of inflammatory cytokines^59^. Our previous study shows TLR7-related gene expression changes already after one R848 injection, suggesting its properties to enter the BBB^17^. However, due to its BBB dysregulatory properties, more R848 might cross the BBB over time in chronic treatment, as performed in this study. Impaired BBB integrity persists after recovery from the primary cause^59,60^, which might be a signature resulting from chronic TLR7 activation, as well. Alcohol-induced neuroimmune alterations, including BBB dysregulation, have been shown to promote drinking^61^. Simultaneously, chronic alcohol consumption leads to long-lasting BBB impairments in humans and animal models of alcohol drinking and dependence, potentially mediated by TLRs^27,62–64^. Together with the alterations in structural and functional BBB regulatory pathways, we also identified alcohol-sensitive genes such as *Pde4b*, *PPP1r1b*, *Grm7* to be differentially expressed in astrocytes. These transcriptomic profiles might link TLR7-induced BBB dysregulation to escalated drinking. However, since this study only profiles indirect transcriptomic signatures of BBB dysregulation and escalated drinking, further studies are required to validate these hypotheses.

The highly conserved Wnt signaling pathways regulate tissue homeostasis and calcium signaling^65^. Genes of the canonical Wnt signaling are expressed in glial and neuronal cells. While in glia, their main role is maintaining BBB plasticity and barrier properties^66,67^, in neurons, Wnt signaling regulates the cytoskeleton and synaptic plasticity. Their involvement in neuronal cell death points towards a role in aging, memory, and neurodegenerative diseases^68,69^. We identified altered genes linking *Tlr7* expression to Wnt signaling, such as *Ifi27* and *Trim59*, suggesting negative regulation of IRF7 inhibition and Wnt signaling. The latter is additionally supported by concordantly down-regulated genes in both, PFC and AMG astrocytes, which are enriched in multiple Wnt signaling pathway terms. Further, IFN signaling genes that are also part of Wnt signaling were differentially expressed in our data (e.g., *Apoe*, *Ddx60*, *Oasl2*, *Ror1*, *Prkd1*). So far, little is known about the interaction between TLRs and Wnt signaling. A recent study on lung cancer found Wnt ligands activating TLR pathways via WNT5A^70^. Furthermore, TLR-induced proinflammatory components are negatively regulated by Wnt/β-catenin signaling^71^.

In the context of alcohol, numerous Wnt signaling genes known to affect alcohol dependent behaviors were found in this study (e.g., *Aldoc*, *Glis3*, *Prkd1*, and *Top2a*). Transcriptomic studies in CIE mice, humans, and binge drinking monkeys, identified Wnt signaling as a strongly enriched pathway, and also partially reported augmented TLR signaling^72–74^. A transcriptome-wide expression study in alcohol exposed mice indicated that altered Wnt signaling may cause neurodegenerative effects^75^. These findings support the non-cell type-specific alterations across multiple brain regions, as seen in our study. These changes might underlie a multi-level mechanism involving genome-wide associations, methylation, and transcription.

Overall, our data suggest TLR7-induced BBB dysregulation through a multilayer mechanism involving astrocyte-specific and non-cell type-specific alterations (Fig. 4). These prolonged impairments may represent a predictive factor for escalated alcohol consumption.

## Methods

All methods were carried out in accordance with relevant guidelines and regulations and are reported in accordance with ARRIVE guidelines.

## Animals

Male C57BL/6J mice (*n* = 28/treatment) aged ~ 7 weeks (Jackson Laboratories, Bar Harbor, Maine, U.S.) were acclimatized to the housing conditions and the experimenters for one week. They were single housed on a 12:12 h light cycle (lights on at 9 am) with unlimited access to food and water. All procedures were approved by the University of Texas Institutional Animal Care and Use Committee and adhered to NIH Guidelines (AAALAC accredited).

### Drug administration and voluntary alcohol consumption

Drug administration was performed as previously described^17^. Briefly, mice were randomly assigned to groups, where they were either treated with 10 intraperitoneal (i.p.) injections of R848 (Resiquimod; 50 µg, dissolved in 1 mg/ml saline; Invitrogen, San Diego, CA, USA) or saline every-other day (EOD) for 20 days in total. R848 selectively activates TLR7 and TLR8 in humans but exclusively TLR7 in mice^76^. 10 days after the last injection, the animals were split into two groups: Group 1 (*n* = 12/treatment) was sacrificed via cervical dislocation immediately after the last treatment-free day, Group 2 (*n* = 16/treatment) was subjected to 8 days (4 drinking days) of 15% EOD 2-bottle choice (2BC) alcohol drinking in their home cages^77^. Water intake was measured daily from the first injection day and throughout the experiment, and body weight was measured weekly (Fig. S1). Escalated drinking was defined as a significant increase in alcohol consumption compared to controls. EOD-2BC drinking was carried to confirm the previously reported R848-induced escalation in alcohol intake^17^. Therefore, this experiment was carried out for 4 drinking days and was aborted after escalation was confirmed. Longer drinking sessions were reported in our previous study^17^ and are not within the scope of this study. Data are represented as g/kg body weight per 24 h, averaged across 2 drinking days. Statistical analyses were performed in GraphPad Prism 10.4.0 (GraphPad Software, Boston, MA, USA) using repeated measures ANOVA with Tukey correction for multiple testing, after confirming homogeneity of variances using Levene’s median test.

## Sample preparation for single nucleus RNA Sequencing (snRNA-Seq)

We performed snRNA-Seq on the mPFC and central AMG (further referred to as AMG) of alcohol-naïve mice repeatedly treated with R848 every-other day for 20 days in total (Fig. 1A, group 1; *n* = 4–5/grp; **Supplementary**). Mice were sacrificed via cervical dislocation and brains were flash frozen in isopentane and stored at -80 °C until further use. The mPFC (prelimbic cortex, Cg1) was dissected and punched between 2.8 and 1.4 mm bregma, and the AMG between (-)0.8- (-)1.8 mm bregma in a cryostat using a 1.5 mm diameter- micro-puncher.

Nuclei were isolated with the Chromium Nuclei Isolation Kit (10X Genomics, Pleasanton, CA) with a reduced lysis time of 4.5 min. snRNA-Seq libraries were prepared utilizing the Chromium Next GEM Single Cell 3’ Kit v3.1 (10X Genomics), and sequenced on a NovaSeq 6000 using an S4 flow cell at the Genomic Sequencing and Analysis Core Facility at UT Austin. A total of 133k cells in the mPFC and 134k cells in the AMG were sequenced.

## Pre-processing of snRNA-Seq data

Fastq files were processed using the CellRanger pipeline (v7.1.0, 10X Genomics) and reads were aligned to the mouse reference genome (mm10, GENCODE v32/Ensembl98) with introns included. CellRanger outputs were imported into R (v4.3.1) using Seurat (v5.1.0.9006)^78^, and SeuratObject (v5.0.2), where one SeuratObject per brain region was generated. Data across all animals per brain region were collapsed using the merge function. Since the animal-specific cluster distribution revealed a homogenous pattern across all animals and consistent representation of animals in all clusters, we decided to not perform further integrational approaches. Nuclei with greater than 10% mitochondrial transcripts were filtered out and mitochondrial and ribosomal genes were removed from the count matrix. Quality control resulted in 132,837 nuclei for mPFC and 129,365 nuclei from AMG. Data were normalized and scaled using SCTransform (v0.4.1)^79^. Nuclei were assigned to cell types by supervised clustering with scSorter (v0.0.2)^80^ and common markers for both brain regions with a balanced number of marker genes per cell type. Supervised subclustering was performed using brain region-specific marker genes (mPFC:^31,32^; AMG:^33,34^. Cell type diversity statistics to identify differences in cell type proportions due to treatment was performed using Speckle^81^.

### Differential gene expression

Differential gene expression analysis was performed using Libra (v1.0.0) to utilize multiple pseudobulking methods within each cell type per brain region. A pseudobulk matrix considering the raw counts per cell type was created and differential gene expression analysis was performed using the three most common approaches for transcriptomic and single cell gene expression datasets: EdgeR, DESeq2, and limma^82^. Differentially expressed genes (DEGs) were defined as a nominal p-value < 0.001 in at least two out of three Differential gene expression approaches. The fold-change calculated by EdgeR is reported and was used for subsequent analyses.

### Gene set enrichment analysis (GSEA)

GSEA was performed per cell type per brain region using the R packages clusterProfiler (v4.10.1) and enrichplot (v1.22.0). Genes were ranked based on the log2 fold-change obtained from the EdgeR differential gene expression analysis described above including all measured transcripts. The ranked gene lists were then tested for significant enrichment in Gene Ontology categories using mouse gene annotations from org.Mm.eg.db (v3.18.0) with Benjamini-Hochberg corrected p-values. For plotting, up- and down-regulated pathways were first converted to -log10(FDR) values, with down-regulated pathways represented as negative values to preserve directionality. GO terms were ranked by absolute cell-type specificity, ignoring direction. Data was combined into one heatmap per brain region, where red indicates up- and blue indicates down-regulated pathways.

### Rank-rank hypergeometric overlap (RRHO) analysis

Rank-Rank Hypergeometric Overlap (RRHO) analysis was performed to identify concordant and discordant transcriptomic signatures in the PFC and AMG in a cell type-specific manner using the R package RRHO2 (v1.0). This approach is based on a “threshold-free” approach to identify genes common across datasets that may be too subtle for traditional stringent significance cut-offs^83^. Ranked gene lists were generated by weighing genes represented in a cell type by each brain regions based on their fold-change determined in the DGE EdgeR analysis and nominal p-value by applying the -log10 of the nominal p-value multiplied by the direction of the log2 fold change. Genes with an average expression of zero in the R848 as well as saline group were removed. The ranked gene lists were then filtered for genes exclusively represented in the respective cell type of both brain regions. RRHO difference maps were produced for group comparisons per cell type by applying the “Stratified method” with step sizes calculated as the square root of the total number of genes in the ranked list. These maps depict concordant genes with similar ranks in both regions in the upper right (down-regulated genes) and lower left box (up-regulated genes) and discordant genes in the upper left and lower right box. The genes closer to the center have a higher rank and red colored spots on the map mean highly significant overlap while blue spots mean low significance. Gene set enrichment analysis of overlapping genes was performed using clusterProfiler (v4.8.3) and the mouse genome org.Mm.eg.db (v3.17.0).

### Cell-cell communication analysis

Cell–cell communication inference was performed using the R package LIANA framework (v1.7.1), which integrates multiple ligand–receptor interaction resources and scoring methods within a unified pipeline. snRNA-seq datasets were analyzed separately for each sample using the rank aggregate consensus scoring strategy implemented in LIANA and CellPhoneDB mouseconsensus interaction database. Cell identities were defined based on the annotated cell-type clusters described above. To enable comparison across samples, ligand–receptor interaction scores were compiled into a multi-sample communication tensor using the tensor-cell2cell framework. The resulting tensor encoded communication scores across ligand–receptor pairs, sender cell types, receiver cell types, and sample contexts. Tensor decomposition implemented in tensor-cell2cell (v0.9.0) was utilized to identify latent communication programs capturing coordinated variation across ligand–receptor interactions, interacting cell types, and samples. The rank of the tensor decomposition was selected based on reconstruction error curves and model stability across multiple random initializations. Factor loadings describing contributions of ligand–receptor pairs, sender cells, receiver cells, and sample contexts were visualized using cluster maps of context loadings and ligand–receptor loadings, heatmaps of joint sender–receiver loading products, and network diagrams summarizing highly weighted communication edges for each factor.

Associations between communication programs and experimental condition were evaluated by comparing sample context loadings between groups using two-sided Welch’s t-tests. Context loadings for each factor were visualized as boxplots with individual sample values overlaid.

To interpret the biological processes associated with each communication program, ligand genes from high-loading ligand–receptor pairs were subjected to gene set enrichment analysis (GSEA). Genes were ranked according to their ligand–receptor loading scores for each factor. GSEA was performed using the R packages clusterProfiler (v4.10.1) and enrichplot (v1.22.0) with Gene Ontology biological processes (GOBP) annotations from org.Mm.eg.db (v3.18.0). Enrichment significance was evaluated using permutation tests via Benjamini–Hochberg correction for multiple hypothesis testing.

### Weighted gene co-expression network analysis (WGCNA)

WGCNA was performed using hdWGCNA (v0.3.03)^84^ with default parameters, a soft power of 6 and minimum module size of 25. Steps of the analysis included gene network construction, module-trait correlation, module eigengene calculation, and hub gene analysis. Treatment was the only variable included for estimating the module eigengene expression.

### Xenium In Situ Spatial Transcriptomics

Spatial Transcriptomics was performed as secondary validation of the cell type-specific gene expression signatures from snRNA-Seq. Coronal Sect. (10 μm) from mice of the same cohort were utilized. Xenium data from AMG-containing sections (Fig. 1A, group 1, *n* = 1/treatment) was acquired using the 10X Genomics Xenium platform with the Prime 5 K Mouse Pan Tissue & Pathways Panel, capturing 5006 pre-designed transcripts. Brain sections at a bregma level of -1.2 mm were sectioned in a cryostat and placed on the Xenium slide. To ensure optimized cell segmentation, the Xenium Multimodal Cell Segmentation Staining was added. Slide processing was performed by the Genomic Sequencing and Analysis Core Facility at UT Austin. Initial quality observation was performed with the 10X Genomics Xenium Explorer 3.2. R (v4.3.1) and Seurat (v4.3.0)^85^ were used for processing of raw counts similar to the description above. DESingle was used for differential gene expression analysis, which uses a Zero-Inflated Negative Binomial model to identify real and dropout zeros to improve accuracy^86^. We extracted nuclear transcripts exclusively to assure comparability with the snRNA-Seq data. Significance threshold for DEGs was set to FDR < 0.05.

## Supplementary Information

Below is the link to the electronic supplementary material.

Supplementary Material 1

## Data Availability

Data is uploaded on the Gene Expression Omnibus data repository (accession number: GSE312659) as well as a Shiny App ( [https://wcaar.shinyapps.io/R848/](https:/wcaar.shinyapps.io/R848) ) and will be publicly available the day of publication onwards. Analysis code is available upon reasonable request.
