# Supplementary material for: Repeated TLR7 activation induces cell type- and brain region-specific transcriptome changes in male mice
Source: Sci Rep. 2026 Apr 30;16:20115. doi: 10.1038/s41598-026-50920-5 (PMC13324148; doi:10.1038/s41598-026-50920-5)
Supplement: Supplementary file 1 — Supplementary Material 1 [file 41598_2026_50920_MOESM1_ESM.docx]

**Supplementary Information**

**Results**

**Repeated TLR7-activation increased alcohol consumption and preference.**

56 mice were administered R848 or saline EOD for 20 days then given 10 days of rest. Following the last day of rest, n=12/treatment were euthanized and n=16/treatment were subjected to 15% EtOH EOD-2BC drinking model (**Fig 1A**). Treatment with R848 caused a significant increase in EtOH intake (**Fig S1A**; main effect of time x treatment; F_(1,30)_=8.615, p=0.0063) and preference for alcohol (**Fig S1B**; main effect of time x treatment; F_(1,30)_=9.130, p=0.0051) but did not affect total fluid intake (**Fig S1C**), compared with saline controls.

**Voluntary alcohol consumption**

EOD-2BC procedure was performed as previously described (Blednov et al. 2011). In brief, two 50 ml canonical tube drinking bottles were presented to animals EOD for 24h, one containing 15% (v/v) EtOH in tap water and one containing water. On off days, mice were given water only. The bottle positions (left/right) were changed each drinking day to account for any preference bias and evaporation estimates were calculated for each mouse from bottles placed in an empty cage. EOD-2BC was carried out for 4 drinking days and was aborted after confirming the escalation of alcohol intake. We ended the behavior here to be able to profile the cell type-specific gene signatures that are promoting the escalation rather than being a consequence of alcohol consumption. Data are presented as g/kg body weight/24h, averaged across 2 drinking days. Statistical analyses were performed in GraphPad Prism 10.4.0 (GraphPad Software, Boston, MA, USA) using repeated measures ANOVA with Tukey correction for multiple testing, after conforming homogeneity of variances using Levene’s median test.


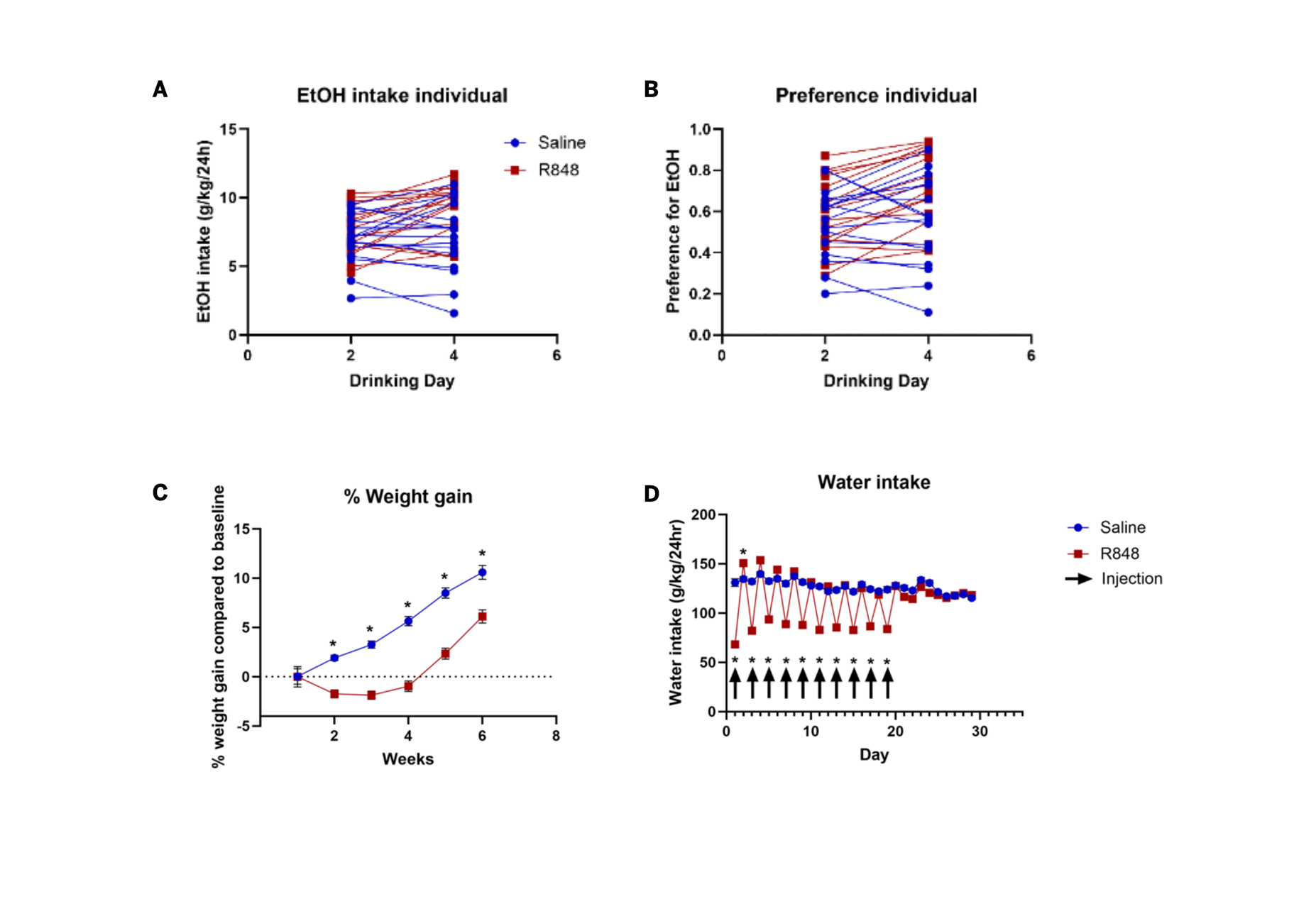


**Supplemental Fig. S1.** **Repeated R848 injections lead to increased ethanol consumption and preference in wildtype mice**. (**A**) Total ethanol consumption was significantly increased in R848-treated mice compared to saline (n=16/group). (**B**) Preference for ethanol was significantly increased in R848-treated mice compared to saline (n=16/group). Administration of R848 also induced acute sickness behaviors, mitigating weight gain (**C**, n=28/group) and decreasing water intake on injection days (**D**, n=28/group). *p.val <0.05 compared to controls ***** p.val <0.05 compared to control group.

**
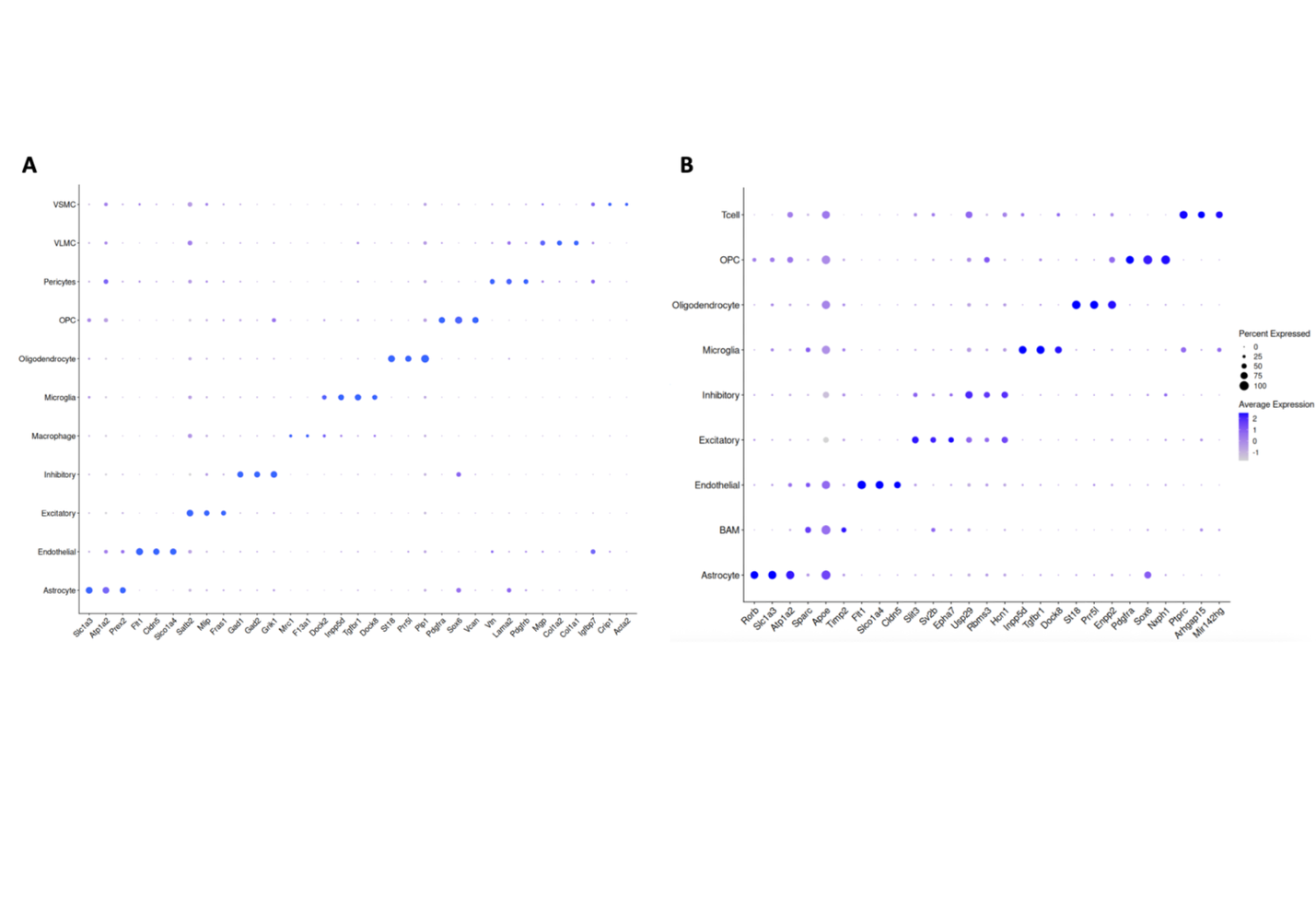
Supplemental Fig. S2**: Top cluster-specific marker genes across all major cell types identified in PFC (**A**) and AMG (**B**).

**Supplemental Fig. S3:** RRHO comparing gene expression profiles from the mPFC and the AMG per cell type. (**A**) RRHO profile of inhibitory neurons capturing 26,513 common transcripts from the mPFC and AMG. The strongest concordant gene expression was found among upregulated genes. (**B**) RRHO profile of excitatory neurons capturing 27,278 common transcripts. The strongest concordant gene expression was found among upregulated genes. (**C**) RRHO profile of astrocytes capturing 23,993 common transcripts. While concordance was found in both up- and down-regulated transcripts, the strongest effects were found among upregulated genes. (**D**) RRHO profile of microglia capturing 21,893 common transcripts. A similar level of concordance was found among both, up- and down-regulated transcripts.

**
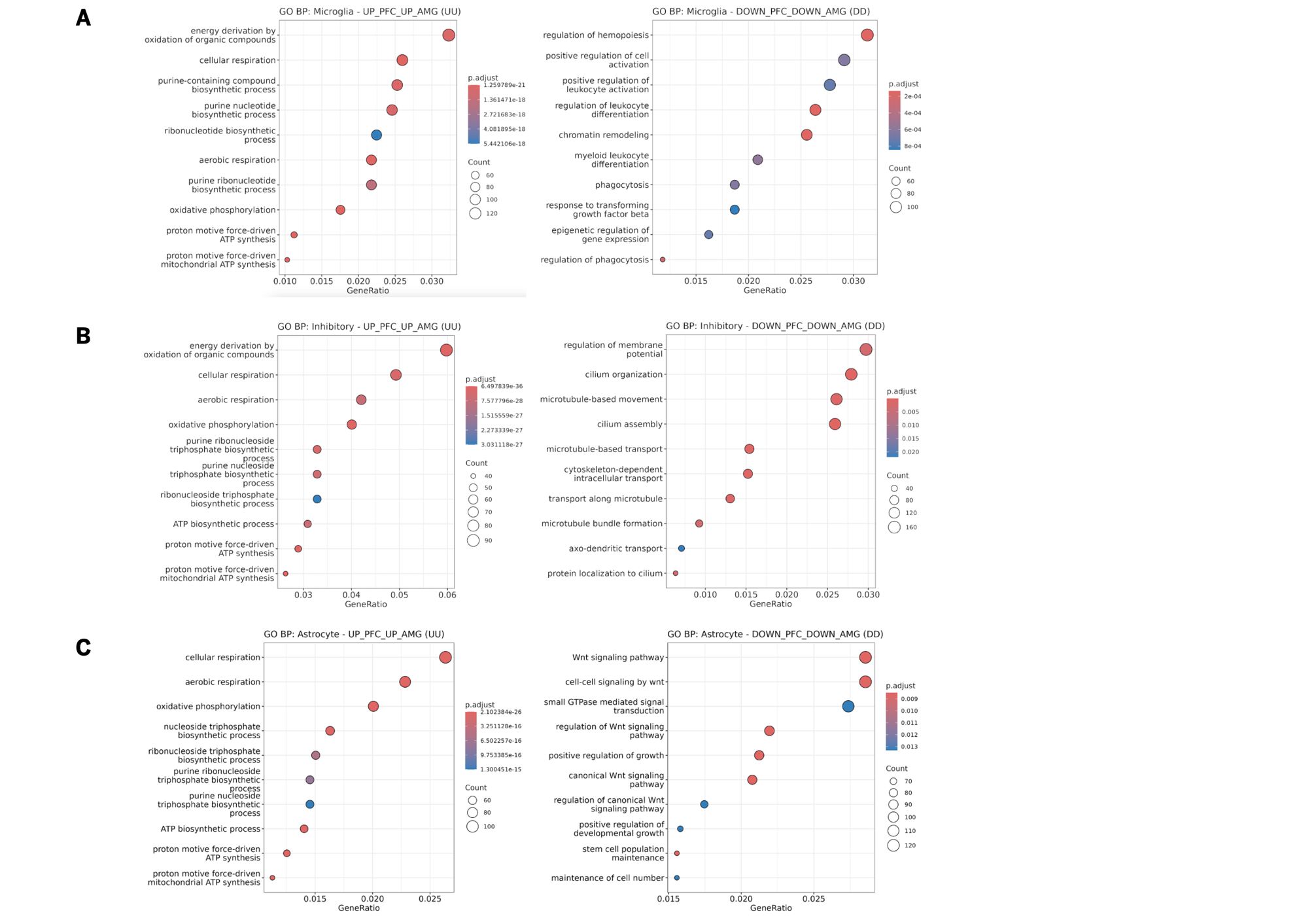
**

**Supplemental Fig. S4:** Gene-set enrichment analysis of concordantly up-and downregulated genes of mPFC and AMG microglia (**A**), inhibitory neurons (**B**), and astrocytes (**C**) resulting from RRHO.


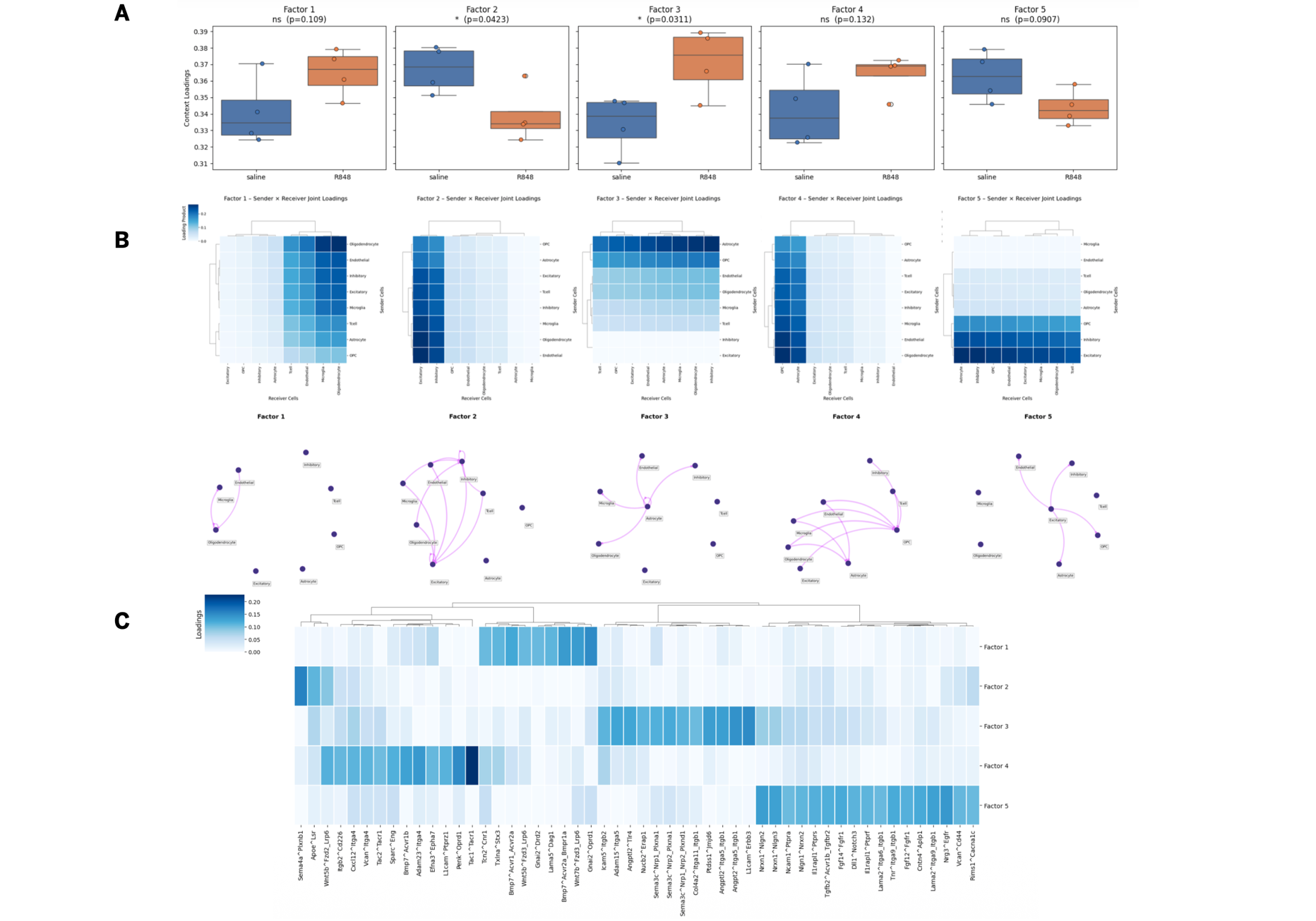


**
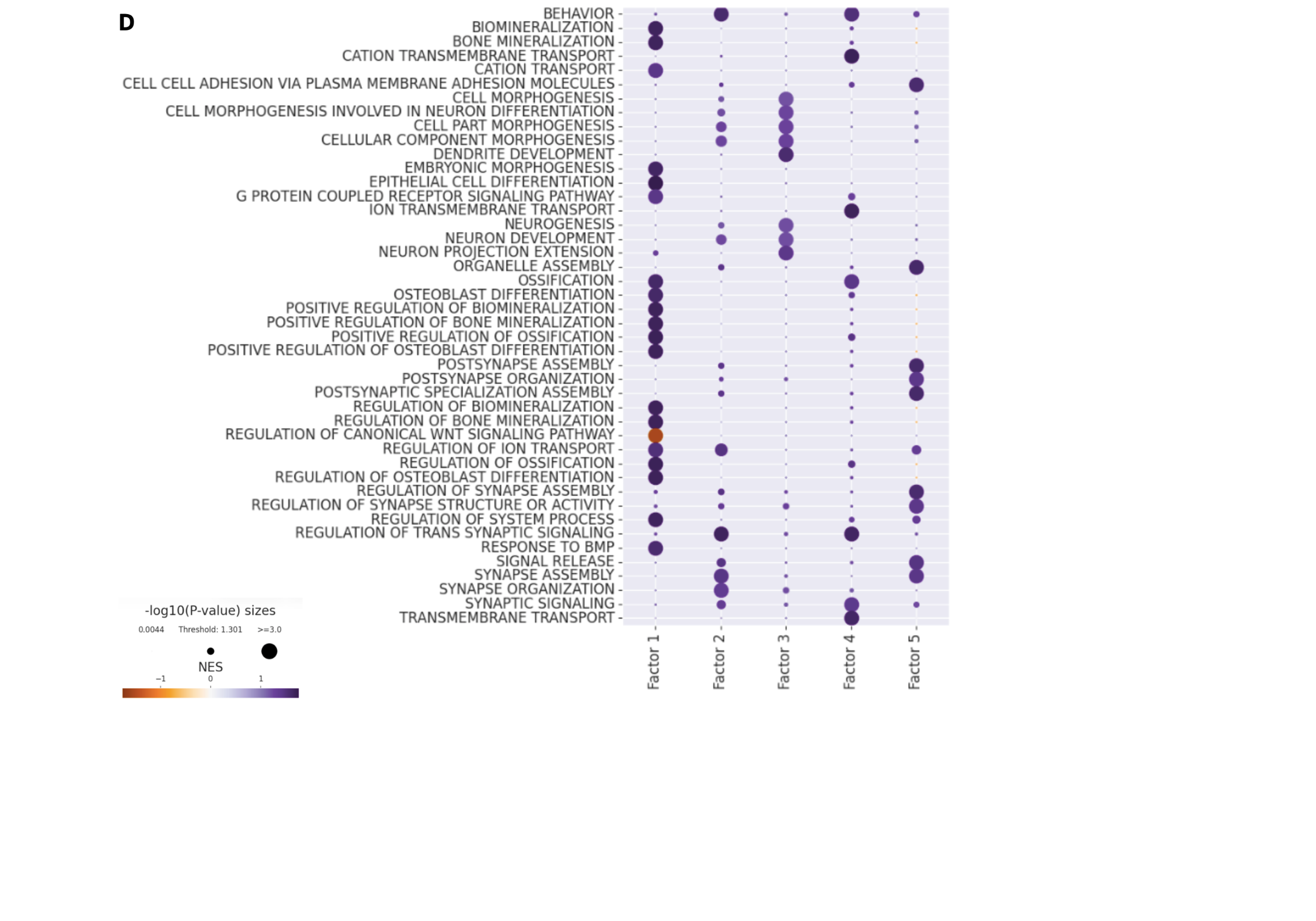
**

**Supplemental Fig. S5:** Cell-cell communication analysis focusing on AMG major cell types. (**A**) Context loadings of communication factors across conditions revealed a significant effect in factor 2 and 3. (**B**) Cell type interaction structure of communication factors show cell type-specific communication per factor. (**C**) Ligand-receptor pairs underlying communication factors by top loading score (threshold = 0.10). (**D**) Gene set enrichment analysis of ligand-receptor pairs per factor. All ligand-receptor pairs were included in this analysis (N=826). Threshold for reported pathways is adjusted p-value < 0.05.


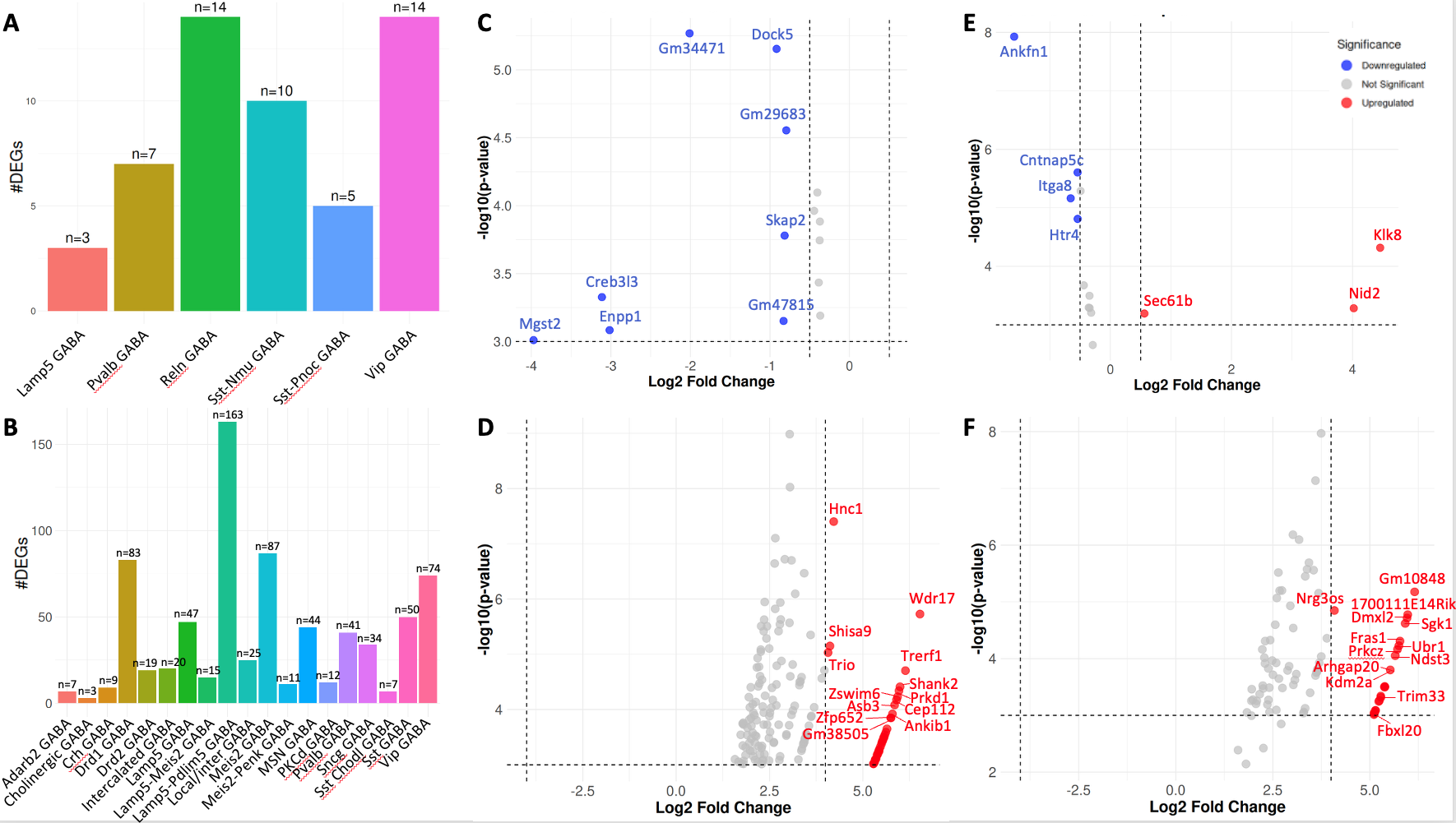


**Supplemental Fig. S6**: Subclustering of inhibitory neurons. (A) Subclustering of PFC inhibitory neurons revealed 6 subtypes and resulted in 53 DEGs with most DEGs in Reln and Vip neurons. (B) Subclustering of AMG inhibitory neurons revealed 19 subtypes and resulted in 751 DEGs with most DEGs in Lamp5-Pdlim5 neurons. (C) Volcano plot of PFC Reln neurons. DEGs with log2 fold-change < +/- 0.5 (dashed lines) are colored in grey. (D) Volcano plot of AMG Lamp5-Pdlim5 neurons. DEGs with log2 fold-change < +/- 3.0 (dashed lines) are colored in grey. (E) Volcano plot of PFC Vip neurons. DEGs with log2 fold-change < +/- 0.5 (dashed lines) are colored in grey. (F) Volcano plot of AMG Meis2 neurons. DEGs with log2 fold-change < +/- 3.0 (dashed lines) are colored in grey.

**Supplemental Fig. S7**: Immune response enrichment analysis of DEGs identified in microglia with macrophage profile of the dictionary of immune responses to cytokines, previously established by Cui et al., Nature (2024). (**A**) PFC microglia expression profiles show a tendency towards enrichment in interleukin responses. (**B**) AMG microglia expression profiles are significantly enriched in interferon responses. IL15 is among the top enriched cytokine responses in both brain regions.

**Supplemental Fig. S8**: Oligodendrocyte and astrocyte subclustering (Tab. S7-10) revealed clusters with blood-brain barrier (BBB) and Wnt signaling transcripts. (**A**) PFC oligodendrocytes subclustering revealed one cluster involved in Wnt signaling markers (cluster 5) and one cluster involved in BBB regulation markers (cluster 4). (**B**) AMG oligodendrocytes subclustering revealed one cluster involved in Wnt signaling markers (cluster 6) and one cluster involved in BBB regulation markers (cluster 4). (**C**) PFC astrocytes subclustering revealed one cluster involved in Wnt signaling markers (cluster 7) and one cluster involved in BBB regulation markers (cluster 1). (**D**) AMG astrocytes subclustering revealed one cluster involved in Wnt signaling markers (cluster 3) and one cluster involved in BBB regulation markers (cluster 4).

**Supplemental Fig. S9**: AMG oligodendrocyte subclustering reveals cluster with microglia marker genes (subcluster 8, Tab. S10). (**A**) UMAP depicting oligodendrocyte subclusters with cluster 8 clustering separately from all other clusters. (**B**) Treatment-specific highlighting of nuclei across all oligodendrocyte subclusters. (**C**) Treatment-specific proportion of nuclei per cluster revealed a slight over-representation of cluster 8 in the R848-treated group. (**D**) Top 15 cluster-specific marker genes of oligodendrocyte subcluster 8 are microglia-specific transcripts. (**E**) UMAP capturing all nuclei of the AMG. Oligodendrocyte subcluster 8 clusters separately from the remaining oligodendrocytes and closer to microglia. (**F**) Microglia subclustering including nuclei from oligodendrocyte subcluster 8. Nuclei from oligodendrocyte subcluster 8 end up in microglia subcluster 3, which clusters separately from most of the remainder microglia subclusters. (**G**) Top 15 cluster-specific marker genes of microglia subcluster 3 are oligodendrocyte-specific transcripts. (**H**) Testing for potential bias of oligodendrocyte subcluster 8 due to doublets. UMAP of oligodendrocyte subclustering highlighting nuclei that were identified as doublet based on the R package DoubletFinder. Doublet percentage across oligodendrocyte subclusters reveal a low (3.11%) percentage of doublets for subcluster 8.


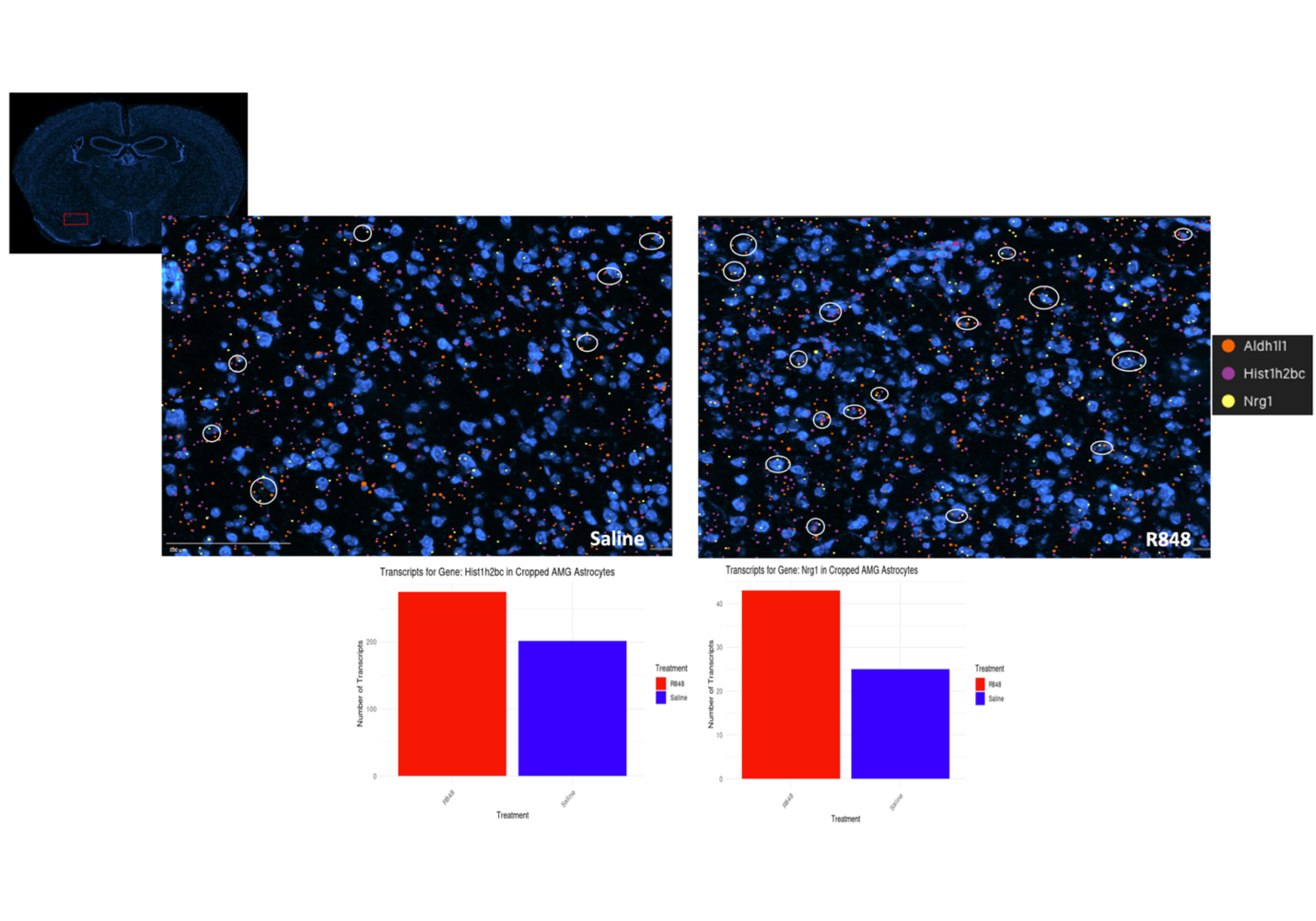


**Supplemental Fig. S10**: Xenium *in-situ* validation of AMG astrocyte DEGs. Upper panel shows a zoomed-in perspective of cells in the AMG crop stained with DAPI (blue). The expression of *Hist11h2bc* (purple) and *Nrg1* (yellow) identified in AMG astrocytes indicated by Aldh1l1 expression (orange) showed increased co-expression of these transcripts. Bottom panel represents the up-regulation of *Hist11h2bc* (left) and *Nrg1* in nuclei of the AMG crop.

**
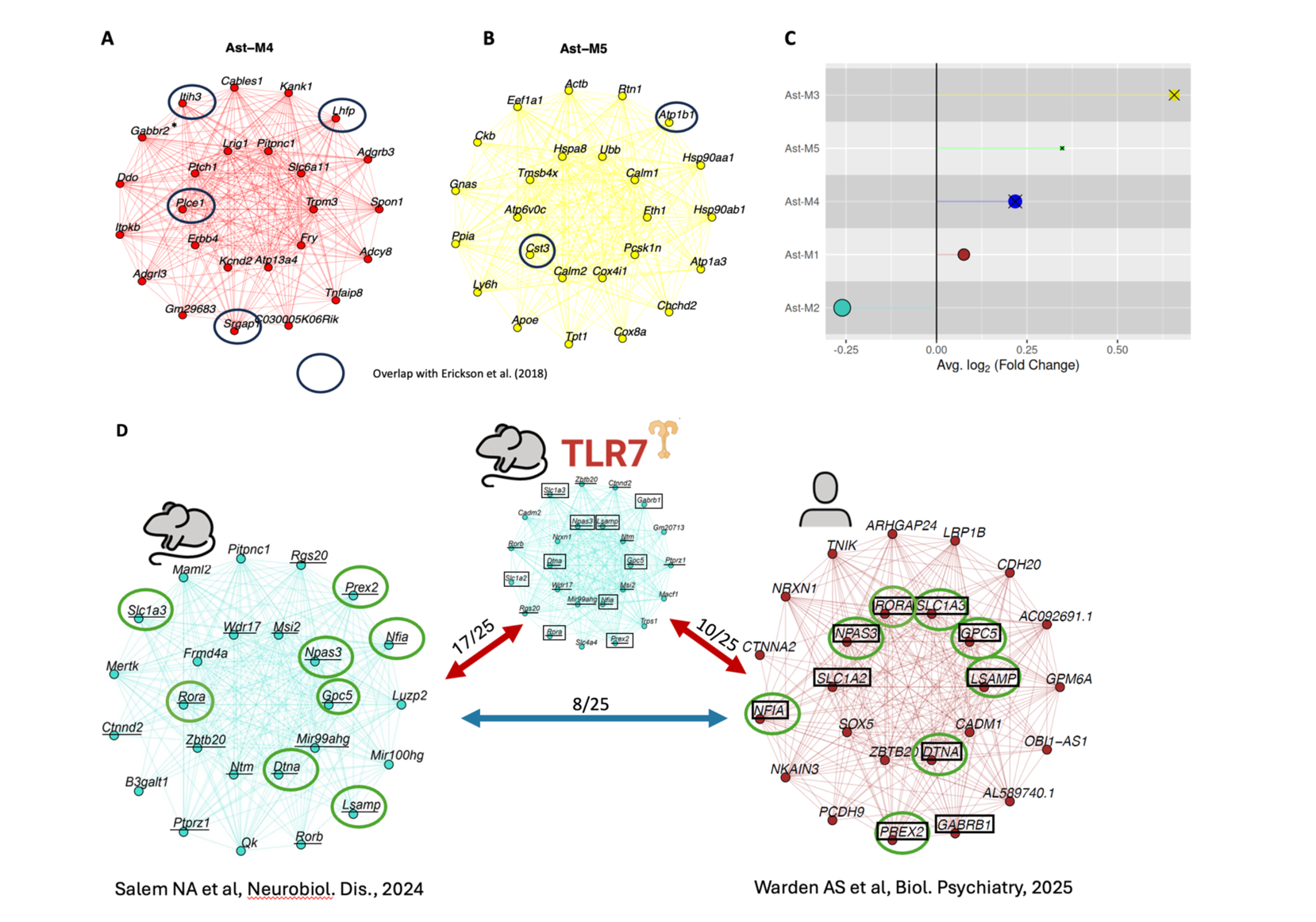
**

**Supplemental Fig. S11**: hdWGCNA analysis in AMG astrocytes. **A, B**) Two modules had overlapping genes with a recently published study on astrocytes enriched from CIE mice (Erickson et al. 2018). **C**) Lollipop plot showing fold change for each of the 5 identified modules; non-significantly changed modules are marked with an X. **D**) One network (Ast-M2) has high similarity with astrocytes of the mPFC of CIE-treated mice (Salem et al., 2024) and astrocytes of the dlPFC of human postmortem brain from individuals with AUD (Warden et al. 2025). Green circles indicate common genes between Salem et al. (2024) and Warden et al. (2025), black underline highlights common genes between the Tlr7 module and the CIE mice from Salem et al. (2024), black boxes indicate common genes between the Tlr7 module and the human AUD analysis from Warden et al. (2025).

**References**

Blednov, Y. A., J. M. Benavidez, C. Geil, S. Perra, H. Morikawa, and R. A. Harris. 2011. “Activation of Inflammatory Signaling by Lipopolysaccharide Produces a Prolonged Increase of Voluntary Alcohol Intake in Mice.” *Brain, Behavior, and Immunity* 25 Suppl 1 (Suppl 1): S92–105.

Cahill, Kelly M., Zhiguang Huo, George C. Tseng, Ryan W. Logan, and Marianne L. Seney. 2018. “Improved Identification of Concordant and Discordant Gene Expression Signatures Using an Updated Rank-Rank Hypergeometric Overlap Approach.” *Scientific Reports* 8 (1): 9588.

Erickson, Emma K., Sean P. Farris, Yuri A. Blednov, R. Dayne Mayfield, and R. Adron Harris. 2018. “Astrocyte-Specific Transcriptome Responses to Chronic Ethanol Consumption.” *The Pharmacogenomics Journal* 18 (4): 578–89.

Govindaraj, Rajiv Gandhi, Balachandran Manavalan, Shaherin Basith, and Sangdun Choi. 2011. “Comparative Analysis of Species-Specific Ligand Recognition in Toll-like Receptor 8 Signaling: A Hypothesis.” *PloS One* 6 (9): e25118.

Grantham, E. K., A. S. Warden, G. S. McCarthy, A. DaCosta, S. Mason, Y. Blednov, R. D. Mayfield, and R. A. Harris. 2020. “Role of Toll-like Receptor 7 (TLR7) in Voluntary Alcohol Consumption.” *Brain, Behavior, and Immunity* 89 (October): 423–32.

Warden, Anna S., Nihal A. Salem, Eric Brenner, Greg T. Sutherland, Julia Stevens, Manav Kapoor, Alison M. Goate, and R. Dayne Mayfield. 2025. “Integrative Genomics Approach Identifies Glial Transcriptomic Dysregulation and Risk in the Cortex of Individuals with Alcohol Use Disorder.” *Biological Psychiatry*, February. https://doi.org/10.1016/j.biopsych.2025.02.895.
